# Mitigating Heat Wave and Exposure Damage to “Cabernet Sauvignon” Wine Grape With Partial Shading Under Two Irrigation Amounts

**DOI:** 10.3389/fpls.2020.579192

**Published:** 2020-11-10

**Authors:** Johann Martínez-Lüscher, Christopher Cody Lee Chen, Luca Brillante, Sahap Kaan Kurtural

**Affiliations:** Department of Viticulture and Enology, University of California, Davis, Davis, CA, United States

**Keywords:** climate change, water stress, shade nets, flavonoids, heat wave, irrigation, fruit exposure, anthocyanin degradation

## Abstract

Rising temperatures in most agricultural regions of the world are associated with a higher incidence of extreme weather events such as heat waves. We performed an experiment to mitigate the impact of heat waves and exposure of berries in grapevine (*Vitis vinifera* cv. “Cabernet Sauvignon”) with untreated vines (Exposed) or with fruit-zone partial shading (Shaded) under 40 and 80% replacement of crop evapotranspiration (ET_*c*_) with sustained deficit irrigation in a factorially arranged experiment. The trial was performed in a vineyard with vertically shoot positioned trellis with a row orientation that concentrated solar radiation exposure on the southwest aspect of the fruit zone. Leaf stomatal conductance (*g*_*s*_) and net carbon assimilation (*A*_*N*_) were significantly lower in shaded leaves under partial fruit-zone shading that resulted in lower pruning mass for Shaded treatments. Stem water potential (Ψ_*stem*_) responded to a large extent to increased irrigation. However, grapevines with partial fruit-zone shading had transiently better water status under 40% ET_*c*_. Cluster maximum temperatures were 3.9°C greater in Exposed grapevines. Exposed clusters had transiently lower acidity and higher pH. However, Exposed clusters on 40% ET_*c*_ had higher total soluble solids (TSS). The experimental vineyard suffered a 4-day heat wave 21 days before harvest, resulting in 25% of the clusters being damaged in Exposed treatment, regardless of irrigation amount. Furthermore, berries in Exposed treatments suffered a great loss of anthocyanins and flavonols even if they were not damaged by direct solar exposure. The pre-planting decision of using a vertically shoot positioned trellis that concentrated solar radiation on the Southwest aspect offered mild protection in a hot climate region with a sunny growing season with extreme heat events during the execution of study. The extreme conditions under which this study was conducted are not unusual, and have become more expected. Our work provided evidence of the vulnerability of grape berry to heat waves and exposure during heat wave events and possible protection methods to mitigate these effects *in situ* in context of climate change.

## Introduction

The commercial success of a grape growing region is based on a fine-tuned match between the climate and cultivar and rootstock selection (Ollat et al., 2016), and it has to be combined with an adequate market demand. By the middle of twenty-first century, climatic conditions are expected to change potentially affecting key physiological and production parameters (Hannah et al., 2013; Fraga et al., 2016). The increase in atmospheric CO_2_ and other greenhouse gasses most certainly will increase the temperature of the planet ranging from 1.5 to 4.5°C (IPCC, 2013). Furthermore, the incidence of extreme events, such as heat waves, is increasing with an associated risk for crops (Fischer and Schär, 2010; Smith, 2011; Deryng et al., 2014; Martínez-Lüscher et al., 2017b). Higher temperatures are associated with greater rates evaporation of water and therefore, higher global precipitation. However, these are unevenly distributed. In fact, most regions where grapevines are grown are forecasted to experience a reduction in cloud coverage and rainfall and an increase in solar radiation reaching the earth’s surface (Trenberth and Fasullo, 2009).

Grapevine is a rather resilient perennial crop, tolerating long periods of drought and extreme temperatures. However, as in many other fleshy fruits, grape berry is sensitive to exposure to solar radiation, causing damage on the surface of the fruit or even fruit abortion (Tinyane et al., 2018; Torres et al., 2020). Fruit exposure to solar radiation was highlighted for decades as a key factor to enhance ripening of fruits and their composition and is a very relevant concept for cultural practices in grapes (Jackson and Lombard, 1993; Cook et al., 2015). Fruit zone leaf removal in dense canopies can promote ripening and synthesis of flavonoids such as anthocyanins and flavonols (Pastore et al., 2013). Under controlled conditions, a combination of visible and UV radiation may upregulate structural and regulatory genes responsible for the synthesis of anthocyanins and flavonols (Azuma et al., 2012). These photomorphogenic effects are mediated by photoreceptors, phytochromes and cryptochromes and are responsive to changes in radiation spectra (González et al., 2015; Matus, 2016). However, solar radiation, especially infrared, transmits thermal energy to exposed objects with an intrinsic increase in their temperature. High temperature may play repressive role in the synthesis of anthocyanins, eventually inducing their degradation (Mori et al., 2007; Martínez-Lüscher et al., 2017b; Torres et al., 2020). In addition, high temperatures may be responsible for a desynchronization between the accumulation of sugars and anthocyanins leading to lower anthocyanin contents at harvest (Sadras and Moran, 2012).

In semiarid climates, where growing season rainfall is not enough to sustain production, grapevines are grown with supplemental irrigation. Mild water deficits may enhance grape ripening, through the re-concentration of the berry contents or improved grape composition through the synthesis of stress-related metabolites (Chaves et al., 2010; Kuhn et al., 2014). However, excessive water deficits in hot climates may lead to deleterious effect on fruit quality (Brillante et al., 2017; Martínez-Lüscher et al., 2017a; Yu and Kurtural, 2020; Yu et al., 2020). High temperatures may exacerbate water deficits by increasing vapor pressure deficit, thus increasing evapotranspiration. Furthermore, an optimal water status during heat events may increase stomatal conductance helping to reduce canopy temperature (Drake et al., 2018). As a consequence, water requirements for a plant under heat stress may be increased (Restaino et al., 2016). Failure to replace enough crop evapotranspiration demand under heat waves may lead to substantial reductions in yields and ultimately crop failure (Terry and Kurtural, 2011; Daryanto et al., 2016). In grapevine, in-season yield loss may be mediated through reductions in berry size, at first, and a severe shriveling and bunch stem necrosis if high water deficits persist (Krasnow et al., 2010; Hall et al., 2011; Cook et al., 2015; Yu and Kurtural, 2020). Furthermore, in hot climates, post-veraison water deficits led to a diminution of anthocyanin content (Martínez-Lüscher et al., 2017a), modulation of anthocyanin profile toward di-hydroxylated anthocyanidins (Yu et al., 2016) with rapid degradation of proanthocyanins of grape berry and wine (Yu et al., 2020).

In our previous research, qualitative and quantitative differences in fruit-zone microclimate were induced by means of color shade nets (Martínez-Lüscher et al., 2017b). We designed an experiment aiming to test the effect of partial solar radiation reduction and irrigation amounts and their interaction on gas exchange, stem water potential, berry temperature, must composition, and skin anthocyanins and flavonols. Specifically, the study aimed to evaluate the vulnerability of “Cabernet Sauvignon” grape berry to heat waves and exposure of clusters to solar radiation; and whether increased water application would palliate either high berry temperatures or deleterious effects on grape composition.

## Materials and Methods

### Experimental Site and Plant Material

The experiment was conducted at the University of California Davis, Oakville Experimental Vineyard (38.428, −122.409; Oakville, CA) during the 2017 growing season. Eight-year old *Vitis vinifera* “Cabernet Sauvignon” Clone FPS08 grapevines grafted on 110 Richter (*Vitis berlandieri × Vitis rupestris*) rootstock were used. Plants were trained to bilateral cordons 0.90 m above vineyard floor (cordon height) and shoots were vertically shoot-positioned on 30-single bud spurs. Vine and row spacing was 2.0 m × 2.4 m, respectively, and rows were oriented Northwest to Southeast. The plants were drip-irrigated with 2 pressure compensating emitters per plant delivering 1.9 L/h each. Air temperatures and reference crop evapotranspiration (ET_*o*_) from 1 March to 31 October for the year of study and the previous 10 years were obtained from the California Irrigation Management Information System network station installed on site. Clear sky days were calculated using the station radiometer and accounting for days with at least 75% to the total radiation ever recorded for that day of the year as reported elsewhere (Martínez-Lüscher et al., 2017b).

### Experimental Design and Treatment Application

The treatments were arranged factorially in a randomized complete block design combining two sustained deficit irrigation amounts, which were 40 and 80% replacement of crop evapotranspiration (ET_*c*_), and the absence/presence of shade nets (Exposed and Shaded). Fractions of ET_*c*_ were calculated weekly from the product of ET_*o*_ and crop coefficient (K_*c*_) as in Williams and Ayars (Williams and Ayars, 2005). The 80% ET_*c*_ was achieved by adding two more emitters (1.9 L/h each) in the corresponding locations on a blind irrigation hose. All irrigations began on 20 May, on the day of 50% flowering and no special measures were taken prior to heat events as the intrinsic increase in ET_*o*_ of high temperatures was calculated and applied at the end of each week. The amount of irrigation applied with the corresponding K_*c*_ is reported in Table 1. The Shade netting that allowed 40% of solar radiation to pass through (60% shading) (Black-40; Ginegar, Kibbutz, Israel) was applied on 15 June, 26 days after flowering (DAF), at 100% fruit set. The shade nets applied as follows: the nets were cut into 6 m (long) × 1 m (wide) strips (Supplementary Figure S1A) and hung onto the Southeast and Northwest sides of the canopy at 0.95–1.95 m above the vineyard floor, 0.25 m above the second catch wire as indicated in Supplementary Figure S1B.

**TABLE 1:** Timing of irrigation amounts for the two treatments applied and parameters used to calculate crop evapotranspiration.

Period accounted	Irrigation (DAF^*a*^)	ET_*o*_^*b*^ (mm)	Precipitation (mm)	Kc^*c*^	40% of ET_*c*_^*d*^ (mm)	80% of ET_*c*_ (mm)
5/13 to 5/19	0	34.1	0	0.21	2.9	5.8
5/20 to 5/26	7	32.9	0	0.32	4.2	8.4
5/27 to 6/2	14	35.2	0	0.39	5.5	11.0
6/3 to 6/9	21	31.9	0	0.43	5.4	10.8
6/10 to 6/16	28	39.7	0	0.46	7.3	14.6
6/17 to 6/23	35	45.6	0	0.57	10.4	20.8
6/24 to 6/30	42	38.1	0	0.57	8.7	17.4
7/1 to 7/7	49	42.5	0	0.52	8.8	17.7
7/8 to 7/14	56	42.0	0	0.57	9.6	19.2
7/15 to 7/21	63	44.0	0	0.52	9.2	18.3
7/22 to 7/28	70	41.3	0	0.54	8.9	17.8
7/29 to 8/4	77	35.9	0	0.51	7.3	14.7
8/5 to 8/11	84	33.5	0	0.51	6.8	13.7
8/12 to 8/18	91	32.5	0	0.51	6.6	13.3
8/19 to 8/25	98	31.1	0	0.51	6.3	12.7
8/26 to 9/1	105	27.2	0	0.51	5.5	11.1
9/2 to 9/8	112	27.6	0	0.51	5.6	11.3
9/9 to 9/15	119	26.4	0	0.51	5.4	10.8
9/16 to 9/22	126	31.8	0	0.51	6.5	13.0
Total		673.3			131.1	262.2

*^a^DAF, Days after flowering. ^b^ET_o_, Reference crop evapotranspiration. ^c^K_c_, Crop coefficient. ^d^ET_c_, Estimated crop evapotranspiration (ET_o_ × K_c_) calculated as reported by Williams and Ayars (2005).*

### Fruit Zone Microclimate

**FIGURE 1:**
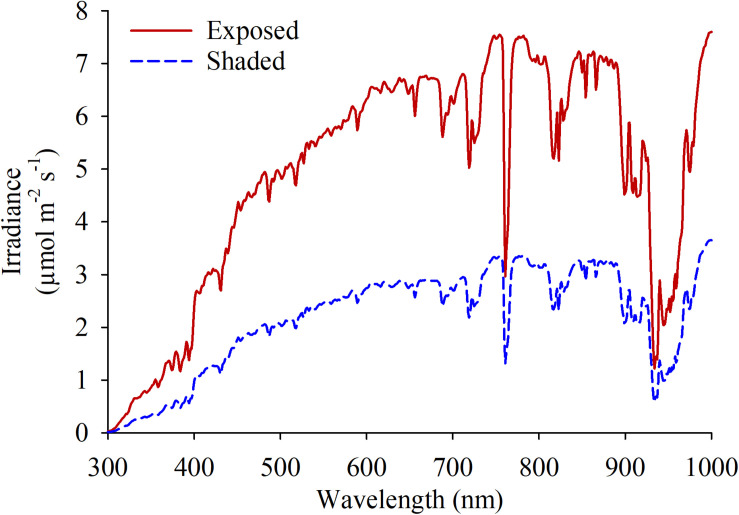
Spectral irradiance measured at the fruit-zone of grapevines fully exposed and shaded at the fruit-zone by Black-40 nets.

Spectral radiation in the fruiting zone were quantified using a spectrometer with a cosine-corrected head (Black Comet-SR, StellarNet; Tampa, FL, United States) for each of the experimental units around 1500 h, which corresponded the hottest moment of the day (Figure 1). One measurement was taken pointing at the sun and 3 additional measurements were taken at 120° rotational intervals to estimate the light coming from every direction. Cluster temperatures were measured as follows. Portable infrared thermometers (Model 2956; Spectrum Technologies; Aurora, IL, United States) were used on berries following onset of anthocyanin accumulation (67 and 114 DAF, respectively) to measure diurnal shifts in cluster temperature. Two clusters with no leaves shading on either aspect of the canopy were chosen and marked in each experimental unit. Three measurements per cluster were averaged every 2 h from sunset until the moment in time when cluster temperature equilibrated with ambient temperature.

### Mid-Day Stem Water Potential

Stem water potential (Ψ_*stem*_) was measured from one fully expanded leaf per vine on the Northeast aspect of the canopy in each plant in the study, 9 times from bud break to harvest. One hour prior to measurement leaves were covered with a re-sealable zip-top foil bag and allowed to equilibrate with stem water potential. Between 1300 and 1400 h local time leaves were excised with a razor blade and measured for water potential with a portable pressure chamber (model 616, PMS Instrument Company, Albany, OR, United States).

### Leaf Gas-Exchange

Leaf gas exchange was measured between 1130 and 1430 h local time 4 times (51, 82, 107, and 113 DAF) throughout the season, using a portable infrared gas analyzer CIRAS-3 (PP Systems, Amesbury, MA), featuring a broad-leaf chamber with 4.5 cm^2^ window. Chamber conditions were set up at 40% relative humidity, a CO_2_ concentration of 400 μmol⋅mol^–1^ and using a flow to the chamber of 300 mL⋅min^–1^. In each experimental unit, one sun-exposed leaf per plant were measured right above the level of nets. In each experimental unit with shade nets, one additional leaf per plant in the lower part of the canopy was measured keeping the shade nets over the cuvette.

### Chemicals and Chromatography Standards

All solvents were of HPLC grade. Chemicals and procedures followed previously described work (Martínez-Lüscher et al., 2019a). Acetonitrile, formic acid, hydrochloric acid and methanol were purchased from Fisher Scientific (Santa Clara, CA). Standards for Malvidin 3*-o-*glucoside, Myricetin-3*-o-*glucoside, Quercetin 3*-o-*glucoside, Quercetin 3*-o-*glucunoride, Quercetin 3*-o-*galactoside, Kaempferol 3*-o-*glucoside, Isorhamnetin 3*-o-*glucoside, and Syringetin 3*-o-*glucoside, obtained from Sigma-Aldrich (St. Louis, MO, United States).

### Sample Collection and Processing

The berries were collected from bunch-closure to harvest. A total of seventy-five berries were collected from each experimental unit on six dates, on both sides of the canopy in equivalent numbers, avoiding severely dehydrated or raisined berries as reported previously (Martínez-Lüscher et al., 2017b; Torres et al., 2020). Each set of seventy-five berries were weighed for individual berry mass calculations. Twenty randomly chosen berries, separate from the 75-berries chosen used in berry composition in section “Berry Composition” (10 from either side of canopy), were weighed and frozen in a −20°C freezer for a minimum of 1 day then individually peeled by hand using a scalpel. Skins were stored a in a −220°C freezer and freeze-dried (Centrivap, Labconco, Kansas City, MO, United States). Dried skins were pulverized in a ball mill (MM400, Retsch, Mammelzen, Germany). A solution of MeOH:H_2_O:7 M HCl (70:29:1) was added to 50 mg of freeze dried, pulverized skin to quantify flavonols and anthocyanins and allowed to extract overnight at 4°C. Following extraction, samples were centrifuged at 14,000 rpm for 10 min and supernatants filtered (0.45 μm; VWR, Seattle, WA, United States) into HPLC vials and analyzed.

### Berry Composition

The 75 berries collected were crushed by hand and filtered to obtain must. A digital refractometer (Palette PR-32, Atago, Tokyo, Japan) was then used to measure total soluble solids (TSS) of filtered juice. Using an autotitrator (862 Compact Titrosampler, Herisau, Switzerland), pH and titratable acidity (TA) were measured. NaOH was used to titrate up to pH 8.2. The TA was expressed as g⋅L^–1^ equivalents of tartaric acid.

### Reversed-Phase High Performance Liquid Chromatography

An HPLC-DAD (1260 series, Agilent, Santa Clara, CA) equipped with a degasser, quarternary pump, thermostatted column compartment and an auto-injector connected to a diode array detector was used to analyze the anthocyanins and flavonols. Mobile phase elution gradient, flavonol and anthocyanin quantification followed previously established procedures (Martínez-Lüscher et al., 2019a) with a reversed phase C18 column LiChrosphere^®^ 100, 250 × 4 mm with a 5 μm particle size and a 4 mm guard column of the same material (Agilent Technologies, Santa Clara, CA, United States). The mobile phase flow rate was 0.5 mL min^–1^, and two mobile phases were used, which included solvent A = 5.5% aqueous formic acid; solvent B = 5.5% formic acid in acetonitrile. The HPLC flow gradient started with 91.5% A with 8.5% B, 87% A with 13% B at 25 min, 82% A with 18% B at 35 min, 62% A with 38% B at 70 min, 50% A with 50% B at 70.01 min, 30% A with 70% B at 75 min, 91.5% A with 8.5% B from 75.01 min to 91 min. The column temperature was maintained at 25°C. Detection of flavonols and anthocyanins was carried out by the diode array detector at 365 and 520 nm, respectively. A computer workstation with Agilent OpenLAB (Chemstation edition, version A.02.10) was used for chromatographic analysis.

Standards for identification of Malvidin 3*-o-*glucoside, Myricetin-3*-o-*glucoside, Quercetin 3*-o-*glucoside, Quercetin 3*-o-*glucunoride, Quercetin 3*-o-*galactoside, Kaempferol 3*-o-*glucoside, Isorhamnetin 3*-o-*glucoside, and Syringetin 3*-o-*glucoside, purchased from Sigma-Aldrich (St. Louis, MO, United States). Malvidin-3*-o-*glucoside and Quercetin-3*-o-*glucoside were used as qualitative standards for anthocyanins and flavonols at 520 and 365 nm, respectively. Other compounds were identified using past literature using mass spectrometry (Castillo-Muñoz et al., 2009; Martínez-Lüscher et al., 2014). Individual anthocyanins and flavonols were grouped by substituents in the 3’, 4’, and 5’ positions of the flavonoid B-ring.

### Yield Components

**FIGURE 2:**
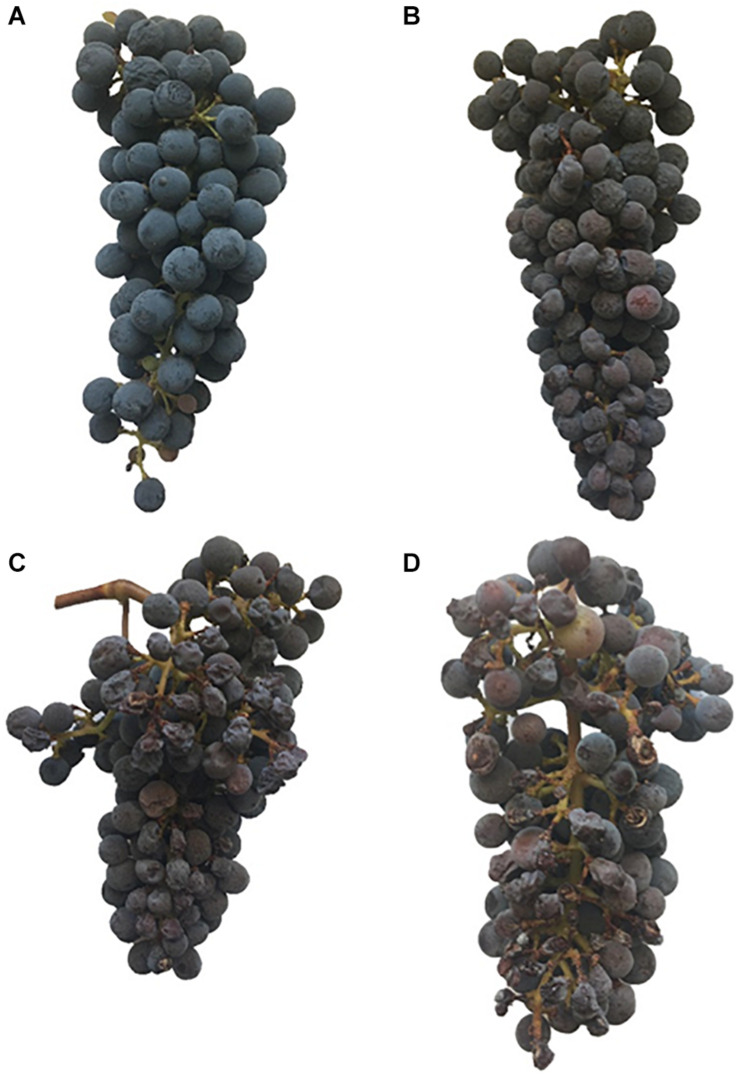
Cabernet Sauvignon (*Vitis vinifera L.)* cluster with no damage **(A)** used for the evaluation at harvest, with mild damage **(B)** some berries discolored and dehydrated, with moderate damage **(C)** many berries discolored, dehydrated, severe damage **(D)** most berries discolored or dehydrated and ripening impaired.

At harvest, clusters were removed, counted, and weighed for each plant in the experiment. At harvest, visible damage to each cluster due to excess solar radiation was quantified on a scale of no damage, mild damage, moderate damage, and severe damage following the subjective criteria presented in Figure 2. In severely damaged clusters, all the grapes in the exposed side of the cluster where completely dehydrated. We measured dormant pruning weights on a top-loading scale, after pruning the grapevines to one bud spurs on 13 February 2018.

### Statistical Analysis

Statistical analysis of data were conducted using SAS v.9.4 (SAS Institute Cary, NC). Data were tested for normality using Shapiro-Wilk’s test and were subjected Levene’s test (Levene, 1960) to ascertain the data met the assumptions of analysis of variance. Percentage of fruit damage data were log-transformed prior to statistical analysis according to results of the Shapiro-Wilk’s test, but non-transformed means are presented to aid in discussion of the corresponding figure. The data were then subjected to a two-way analysis of variance (ANOVA). When the results of ANOVA were significant at *p* < 0.05 data were then subjected to *post hoc* Tukey’s HSD test.

## Results

### Environmental Conditions and Heat Waves

**FIGURE 3:**
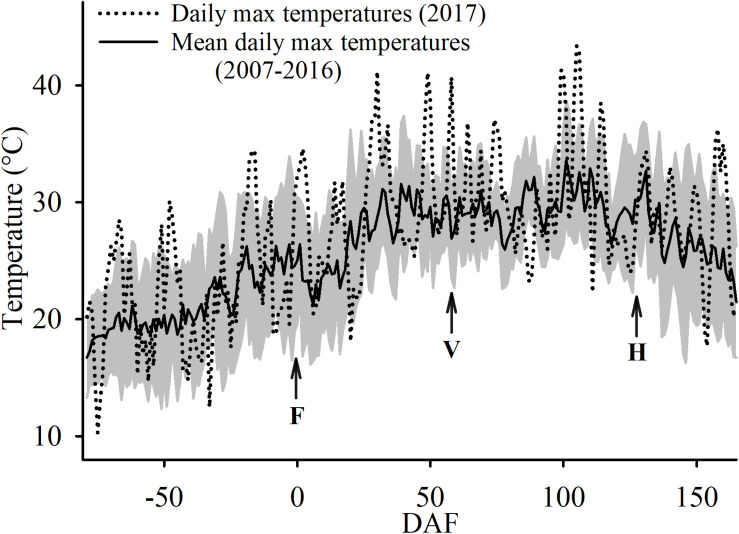
Ten-year average (± SD; shaded area) and 2017 maximum air temperatures at the Oakville Experimental Vineyard as recorded. F, for the date of 50% flowering; V, for 50% veraison and H, for commercial harvest.

During execution of the trial, 80% of the days had no cloud cover and were sunny. The conditions of no cloud cover and sunny days was similar to the conditions during the last 10 years at the study site. However, the growing season in which the experiment was conducted was slightly warmer. The average daily maximum temperature was 1.5°C warmer 2017 (28.2°C) when compared to the last 10 years’ average, at 26.7°C. Furthermore, the experimental year had several heat wave events where maximum air temperatures above 40°C that were recorded on 18 June (29 DAF), 7 July (48 DAF), 16 July (57 DAF), 26 August (98 DAF), 27 August (99 DAF), 1 September (104 DAF), and on 2 September (105 DAF) (Figure 3). In fact, on 2 September the maximum air temperature was 43.4°C which was the absolute maximum temperature recorded at the study site since 2006.

### Plant Water Status

**FIGURE 4:**
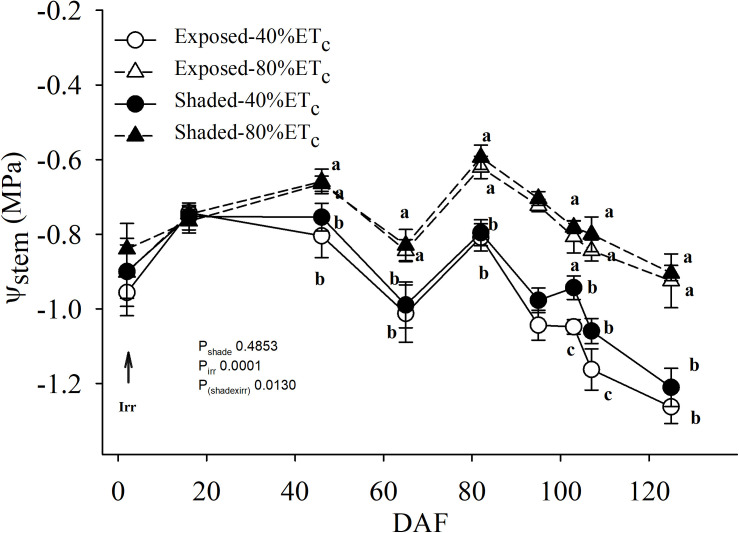
Mid-day stem water potential (Ψ_*stem*_) of plants Exposed or 60% Shaded by nets at the fruit zone level combined with two applied water amounts, 40 and 80% replacement of crop evapotranspiration (ET_*c*_). Error bars represent standard errors (*n* = 4). Groups with no letters in common are statistically different (*p* < 0.05).

Irrigation was not initiated until 20 May 2017 (Table 1 and Figure 4). There was no effect of partial shading on stem water potential throughout the monitoring period (Figure 4). The applied water amounts for the 40%ET_*c*_ treatment were half of the 80%ET_*c*_ treatment as planned (Table 1). The stem water potential of 80% ET_*c*_ treatments started to be significantly different from 40% ET_*c*_ on 65 DAF. The differences of stem water potentials between the 40 and 80% ET_*c*_ treatments increased throughout the season, culminating at harvest, when 40% ET_*c*_ had *ca*. −1.23 MPa and 80% ET_*c*_ had −0.90 MPa.

### Leaf Gas Exchange and Microclimate

**FIGURE 5:**
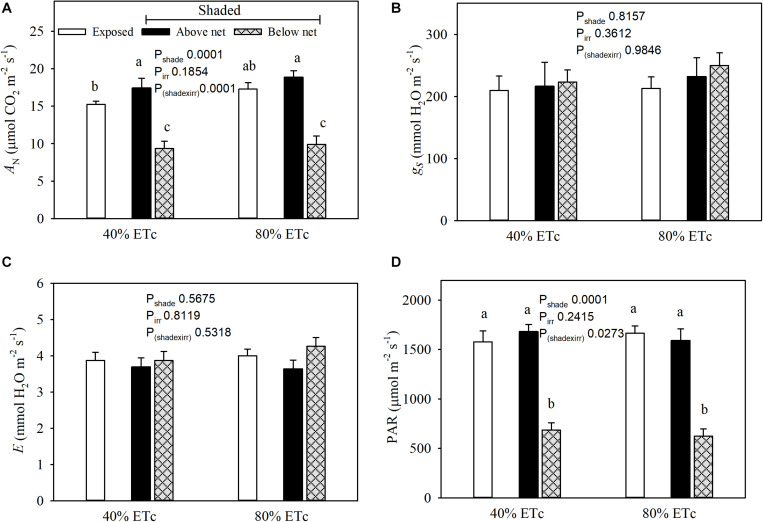
Seasonal averages of leaf gas exchange parameters (Net carbon assimilation, **A**; stomatal conductance, **B**; and evapotranspiration, **C**) and chamber PAR **(D)** measured in plants Exposed or 60% Shaded by nets at the fruit zone level combined with two applied water amounts, 40 and 80% replacement of crop evapotranspiration (ET_*c*_). For plants with fruit zone shade nets, leaves above and below the fruit-zone shade were measured. Error bars represent standard errors (*n* = 12). Groups with no letters in common are statistically different (*p* < 0.05).

Net carbon assimilation (*A*_*N*_) was reduced in leaves under the partial shading (Figure 5A). This was associated with a strong decrease in incident *PAR* of nearly 60% (Figure 5D). Conversely, the leaves above the nets with 80% ET_*c*_ had greater *A_*N*__*t*_* than Exposed–40% ET_*c*_. The 80% ET_*c*_ treatment did not display higher stomatal conductance (*g*_*s*_) or leaf evapotranspiration (*E*) (Figures 5B,C) under our conditions.

### Cluster Temperature

**FIGURE 6:**
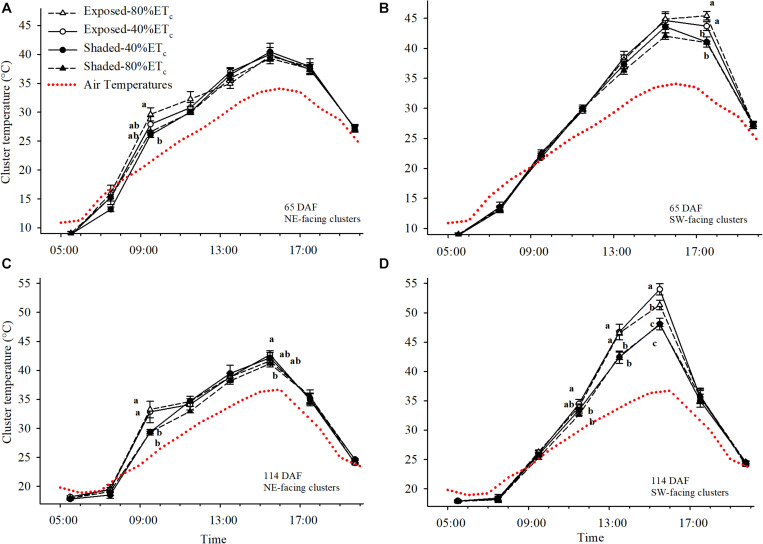
Cluster and air temperatures measured on 24 July **(A,B)** and 11 September **(C,D)** in plants Exposed or Shaded by nets at the fruit zone level combined with two applied water amounts, 40 and 80% replacement of crop evapotranspiration (ET_*c*_). Error bars represent standard errors (*n* = 4). Groups with no letters in common are statistically different (*p* < 0.05). The Northwest and Southwest aspects of the canopy were assessed.

We initially determined the cluster temperatures at 65 DAF, during immediate pre-veraison (Figures 6A,B). The air temperature reached a maximum of 33.5°C. Under these conditions, Exposed-80% ET_*c*_ Northeast-facing clusters started to warm up significantly above air temperature (up to 8°C with Exposed 80%-ETc at 0930 h) and reached 29.6°C, which was 3.5°C higher than the partially Shaded clusters (Figure 6A). However, after 1100 h solar radiation no longer reached Northeast-facing clusters and cluster temperatures tended to equilibrate with air temperature. Conversely, Southwest-facing clusters at 65 DAF started to be fully exposed to solar radiation after 1300 h and cluster temperatures started to separate among Exposed and Shaded clusters (Figure 6B). However, significant differences were not observed until 1730 h, when Exposed–40%ET_*c*_ and Exposed–80% ET_*c*_ reached 45.4°C, which was approximately 4.5°C greater than the Shaded clusters.

At 114 DAF, when the second cluster temperature measurements were collected; clusters were more fully colored, and the air temperature reached 38°C. This resulted in cluster temperatures reaching 33.2°C at 1130 h in Northeast-facing Exposed clusters. These clusters were 3.85°C hotter than the Shaded (Figure 6C). Differences were more pronounced in the Southwest-facing Exposed clusters that were 4°C warmer than the Shaded clusters at 1330 h. Furthermore, cluster temperature of Exposed–40%ET_*c*_ clusters were nearly 6°C higher than the Shaded clusters at 1530 h (Figure 6D). Increasing the irrigation amount to 80%ETc provided some relief to Exposed clusters, albeit the cluster temperatures of Exposed–80% ET_*c*_ were 9°C greater than the ambient air temperature.

### Berry Fresh Mass and Must Composition

**FIGURE 7:**
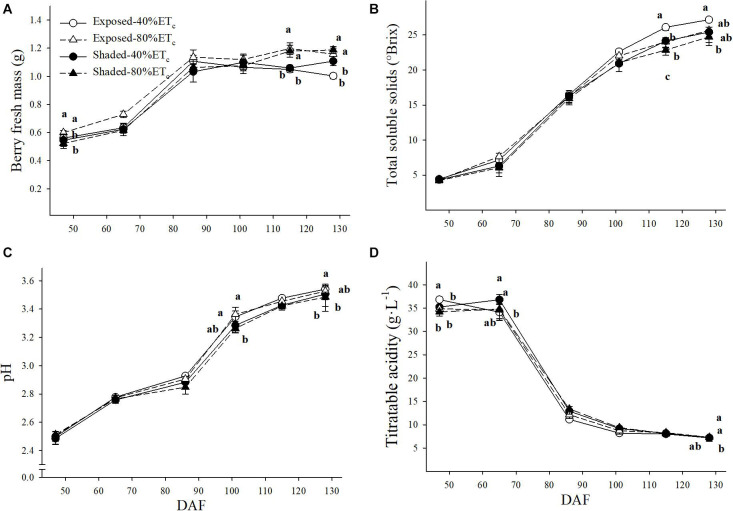
Berry fresh mass **(A)**, must total soluble solids **(B)**, pH **(C)** and titratable acidity **(D)** in plants Exposed or Shaded 60% by nets at the fruit zone level combined with two applied water amounts, 40 and 80% replacement of crop evapotranspiration (ET_*c*_). Error bars represent standard errors (*n* = 4). Groups with no letters in common are statistically different (*p* < 0.05).

Significant differences in berry fresh mass (BFM) were intermittent as 80% ET_*c*_ tended to have greater BFM throughout the growing season (Figure 7A). For instance, the BFM of Exposed–80%ET_*c*_ BFM was greater than the rest of the treatments at 65 DAF. At 115 DAF, BFM was higher in 80% ET_*c*_ treatments regardless of the presence of Shading. However, at harvest (128 DAF), only the BFM of Shaded–80%ET_*c*_ was higher than the rest of the treatments. TSS were lower with Shaded-80% ET_*c*_ at the two last berry samplings (Figure 7B). The Exposed–40%ET_*c*_ had the greatest TSS (27.2°Brix) and Shaded–80%ET_*c*_ the lowest TSS (24.7°Brix) at harvest. Similar intermittent significant differences were observed for pH and titratable acidity (TA) as BFM. The Exposed–40%ET_*c*_ had higher pH (Figure 7C) and lower TA (Figure 7D) than Shaded treatments at 86 DAF. We observed a similar trend at 102 and 115 DAF, where TA and pH were significantly different, respectively.

### Berry Skin Anthocyanins and Flavonols

**FIGURE 8:**
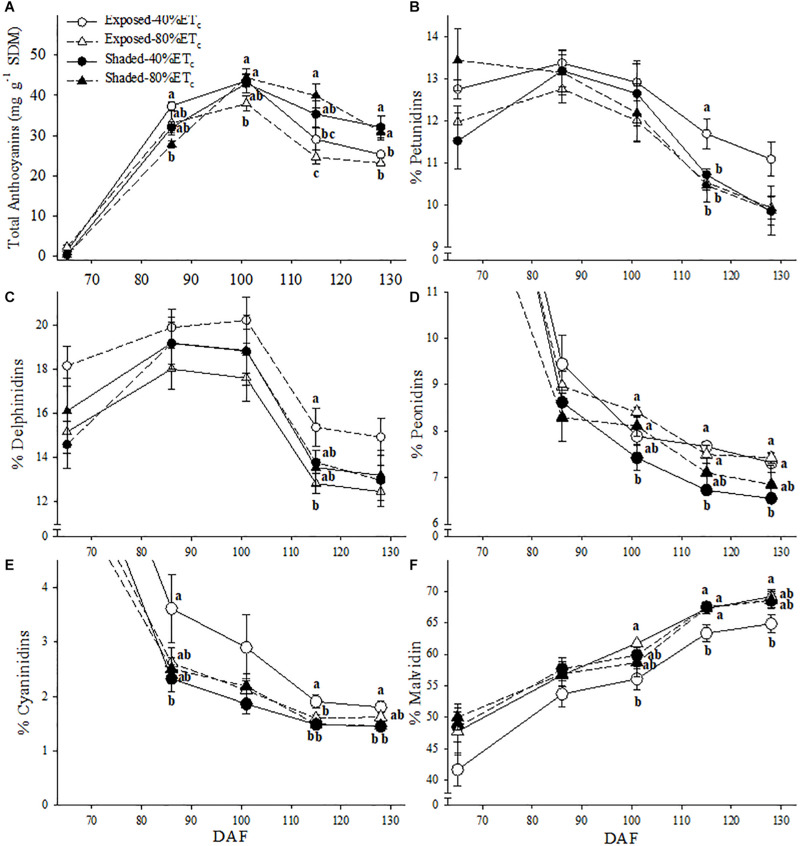
Berry skin anthocyanin content **(A)** and the relative abundance petunidin **(B)**, delphinidin **(C)** peonidin **(D)** cyanidin **(E)** and malvidin **(F)** of in plants Exposed or Shaded 60% by nets at the fruit zone level combined with two applied water amounts, 40 and 80% replacement of crop evapotranspiration (ET_*c*_). Error bars represent standard errors (*n* = 4). Groups with no letters in common are statistically different (*p* < 0.05).

Berry skin anthocyanins were initially greater in Exposed–40% ET_*c*_ at 86 DAF (Figure 8A). However, as all treatments reached their maximum anthocyanin concentration, it started to decrease in the Exposed–40% ET_*c*_ treatment. By harvest (128 DAF), Exposed treatments, regardless of irrigation treatment had lower anthocyanins than the Shaded treatments. Shading and irrigation appeared to have an additive effect on the anthocyanin profile. This effect revealed itself as a decreases in the proportion of petunidin (Figure 8B), and cyanidin (Figure 8E) in favor of malvidin (Figure 8F). This effect was more pronounced especially toward harvest. Furthermore, the proportion of peonidin was only lower in the Shaded–40%ET_*c*_ for the last 3 sampling points (Figure 8D).

**FIGURE 9:**
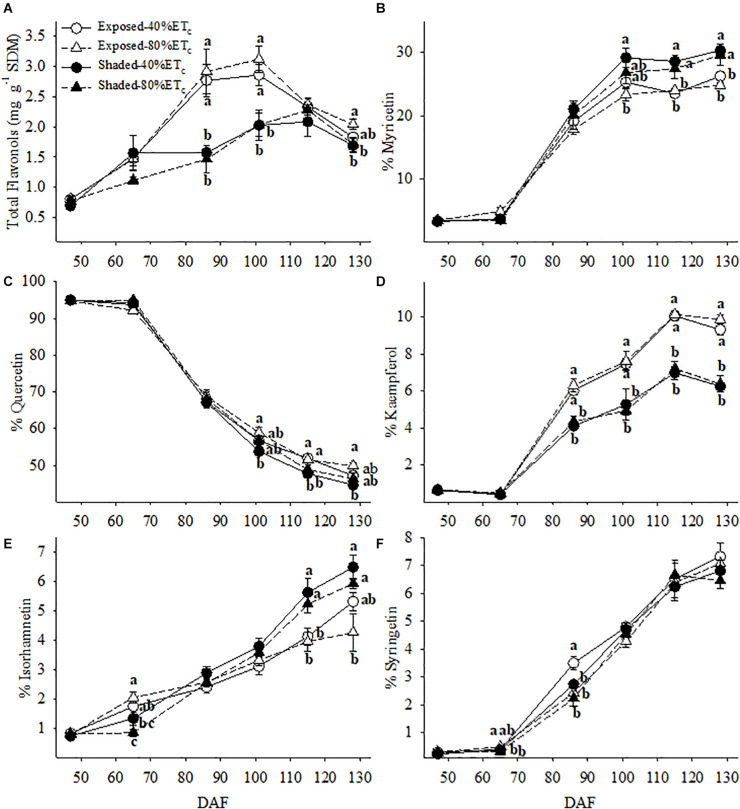
Berry skin flavonol content **(A)** and the relative abundance of myricetin **(B)**, quercetin **(C)**, kaempferol, **(D)**, isorhamnetin **(E)** and syringetin **(F)** in plants Exposed or Shaded 60% by nets at the fruit zone level combined with two applied water amounts, 40 and 80% replacement of crop evapotranspiration (ET_*c*_). Error bars represent standard errors (*n* = 4). Groups with no letters in common are statistically different (*p* < 0.05).

Berry skin flavonol concentration was more than 2 times greater in the Exposed berries at 86 and 102 DAF (Figure 9A). However, this difference decreased as flavonol concentration increased in the Shaded berries until 115 DAF. Meanwhile the Exposed berries reached their maximum flavonol concentration at 86 DAF. Flavonol profile was in most cases affected by the presence or absence of Shading rather than additional irrigation amounts. The proportion of myricetin and isorhamnetin was greater in the Shaded treatments for the last three berry sampling points (Figures 9B,E). Likewise, this corresponded to decreases in the proportion of quercetin (Figure 9C). The proportion of kaempferol was lower in Shaded treatments and this difference continued to increase through harvest regardless of irrigation amount (Figure 9D).

During the most severe heat wave event of the year (103–106 DAF), anthocyanin loss occurred both in Exposed and Shaded berries. However, the loss was more pronounced in the Exposed berries (Table 2). We measured significant changes in the anthocyanin composition of berries which revealed themselves as reductions in the proportion of delphinidins, mainly in favor of malvidin in the Exposed treatment. Conversely, this modulation of anthocyanin profile was not observed in the Shaded berries. Likewise, flavonols in the Exposed treatment experienced a loss, as well. However the flavonol losses were less severe than the anthocyanins. During this most severe heat wave event, we measured a modulation of the flavonol profile, as well. After the heat wave, the proportion of kaempferol and laricitin were greater in detriment of myricetin.

**TABLE 2:** Effects of a 4-day-long heat event with a maximum temperature of 43.4°C on anthocyanins and flavonols.

	Exposed			Shaded			% Change^*a*^		
	Before	After	*p-*value	Before	After	*p-*value	Exposed	Shaded	*p-*value
TA^*b*^ (mg g^–1^ SDM^*c*^)	40.8	28.6	***^d^	43.7	34.9	**	−29.8	−20.2	n.s.
TA (mg per berry)	2.28	1.43	***	2.24	1.74	**	−37.1	−22.0	*
TA (mg g^–1^ BFM^*e*^)	2.09	1.30	***	2.06	1.58	***	−37.8	−23.2	*
3′4′-OH									
% Cyanidins	2.51	2.15	n.s.	2.02	2.02	n.s.	−0.4	0.0	n.s.
% Peonidins	8.15	8.08	n.s.	7.77	7.95	n.s.	−0.1	0.2	n.s.
3′4′5′-OH									
% Delphinidins	18.91	16.44	*	18.83	17.45	n.s.	−2.5	−1.4	n.s.
% Petunidins	12.46	11.90	n.s.	11.17	12.04	n.s.	−0.6	0.9	n.s.
% Malvidins	57.97	61.43	n.s.	60.21	60.54	n.s.	3.5	0.3	*
TF^*f*^ (mg g^–1^ SDM)	3.05	2.52	*	2.10	2.21	n.s.	−17.5	5.1	n.s.
TF (mg per berry)	0.17	0.13	**	0.11	0.11	n.s.	−26.1	2.2	n.s.
TF (mg g^–1^ BFM)	0.16	0.11	***	0.10	0.10	n.s.	−26.7	0.7	n.s.
4′-OH									
% Kaempferol	7.37	9.84	***	4.93	6.90	n.s.	2.5	2.0	n.s.
3′4′-OH									
% Quercetin	56.60	56.93	n.s.	53.50	56.84	n.s.	0.3	3.3	n.s.
% Isorhamnetin	2.10	2.29	n.s.	2.38	2.59	n.s.	0.2	0.2	n.s.
3′4′5′-OH									
% Myricetin	25.87	22.12	***	30.34	24.51	n.s.	−3.7	−5.8	n.s.
% Laricitin	2.56	3.04	**	3.19	3.33	n.s.	0.5	0.1	n.s.
% Syringetin	5.49	5.79	n.s.	5.66	5.84	n.s.	0.3	0.2	n.s.

Total anthocyanins, total flavonols and the relative abundance of each group on 30 August (before the heat wave and 102 DAF) and 3 September (after the heat event and 106 DAF) in plants exposed or 60% shaded by nets at the fruit zone level. Two applied water amounts were pooled together (n = 8). ^a^Percent of change of each variable before and after the heat wave to compare the loss of flavonoids in Exposed and Shaded berries. ^b^TA, Total anthocyanins. ^c^SDM, Skin dry mass. ^d^Analysis of variance p-values stand for “n.s.” for p > 0.05, “*” for p < 0.05, “**” for p < 0.01 and “***” for p < 0.001. ^e^BFM, Berry fresh mass. ^f^TF, Total flavonols.

### Yield Components and Heat Wave Damage at Harvest

Harvest commenced at 128 DAF. The cluster weight (Table 3) was slightly greater with the 80% ET_*c*_. However, this did not result in higher yield per grapevine. We measured a 15% decrease of dormant pruning mass in the Shaded treatment, and this resulted in a greater yield-to-pruning mass ratio. However, the yield-to-pruning mass ratio was not statistically different.

**TABLE 3:** Effects of partial solar radiation exclusion by Black-40 nets and replacement of 40% or 80% of estimated crop evapotranspiration replacement by irrigation on components of yield Cabernet Sauvignon grapevines.

	Cluster no.	Cluster weight (g)	Yield (kg/plant)	Dormant pruning mass (kg/plant)	Ravaz index^*a*^ (kg/kg)
Exposed–40%ET_*c*_	60.6 ± 3.8	102.1 ± 2.4	6.2 ± 0.5	1.49 ± 0.1	4.2 ± 0.1
Exposed–80%ET_*c*_	59.3 ± 1.3	120.5 ± 6.3	7.2 ± 0.5	1.47 ± 0.0	5.0 ± 0.3
Shaded–40%ET_*c*_	57.1 ± 3.8	106.5 ± 7.1	6.1 ± 0.7	1.20 ± 0.1	5.3 ± 0.7
Shaded–80%ET_*c*_	55.8 ± 0.9	113.8 ± 6.4	6.3 ± 0.3	1.30 ± 0.1	5.2 ± 0.4
*P(shade)*	n.s.^*b*^	n.s.	n.s.	*	n.s.
*P(irr)*	n.s.	*	n.s.	n.s.	n.s.
*P(Shade × irr)*	n.s.	n.s.	n.s.	n.s.	n.s.

^a^Ravaz index = Ratio of fruit weight (kg) to pruning weight (kg). ^b^Two-way ANOVA p-values of main factors and their interaction: as “n.s.” for p > 0.05, “*”.

**FIGURE 10:**
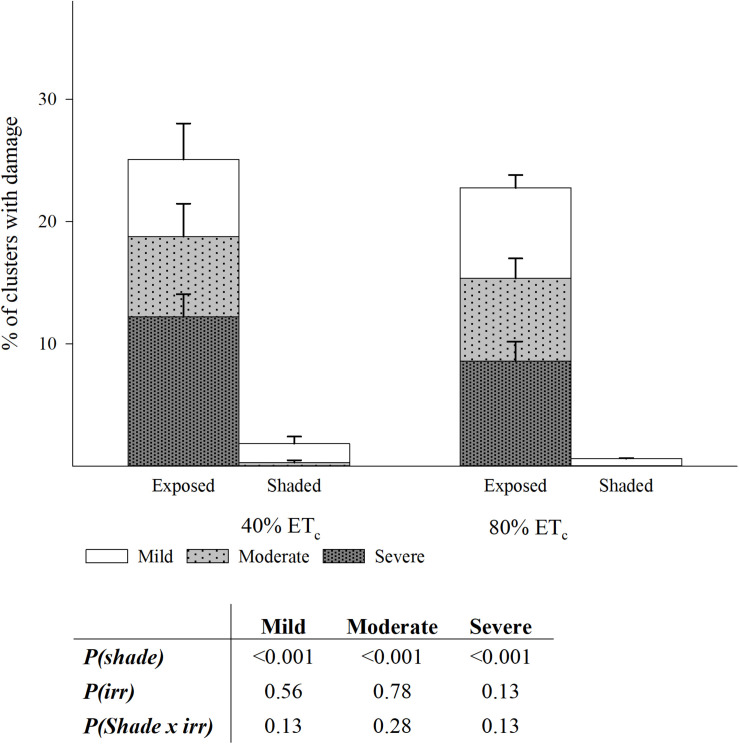
Percentage of clusters with three levels of damage in plants Exposed or Shaded 60% by nets at the fruit zone level combined with two applied water amounts, 40 and 80% replacement of crop evapotranspiration (ET_*c*_). Error bars represent standard errors (n = 4). Cut off sampling points did not present statistical differences. Table with two-way ANOVA *p*-values of main factors and their interaction for each of the damage categories.

The Southwest-facing clusters in the Exposed treatments showed evident signs of damage consisting in desiccated and aborted berries, affecting the appearance of clusters to a different extent (Supplementary Figure S2). In fact, the greater part of the berry abortion happened immediately after a heat wave with temperatures of 43.4°C and 42.8°C on 104 and 105 DAF, respectively. The proportion of damaged clusters was greater in the Exposed treatments when compared to Shaded (24 vs. 2%; Figure 10). Out of this damage, more than a third was severe; indicating that all the visible portions of the grape cluster, directly exposed were completely damaged. The 40%ET_*c*_ treatments had slightly higher proportion of damaged clusters than 80%ET_*c*_ but these were not significantly different. First signs of berry damage were observed 89 DAF on 17 August as a mild discoloration of the grape skins most oriented toward the sun only in well-exposed clusters on the Southwest aspect of the canopy. This damage progressed into flesh tissue death and berry mummification over the course of weeks (Supplementary Figures S2E,F). However, a heat shock phenomenon occurred the days of the heat wave, where sun-facing grapes within a cluster would discolor within hours and mummify over 1 or 2 days (Supplementary Figures S2A,B,G). A third phenomenon, appeared by harvest where whole clusters were found to be completely mummified regardless of their exposure. Previously, this phenomenon was referred to as bunch stem necrosis (Krasnow et al., 2010).

## Discussion

### Environmental Conditions and Heat Waves

During the execution of this study, a 4-day heat wave affected the study site from 31 of August to the 3 of September (103-106 DAF). Although other high heat events occurred that year and the preceding years (Koch et al., 2012; Martínez-Lüscher et al., 2017b) at the study site, it was undeniable that this 4-day period with temperatures above 37°C with a maximum temperature of 43.4°C conditioned our results. These temperatures have not been observed at the study site since 2006. Therefore, results can be interpreted in the context of dealing with extreme climatic events that may become more frequent in many viticultural regions in the context of climate change predictions (Dosio et al., 2018). In regions that are accustomed to growing wine grapes under such high temperatures (Kurtural et al., 2013; Martínez-Lüscher et al., 2017a) events such as the one witnessed here are buffered with the use of different trellising systems. However, with the pre-planting decision of a vertical shoot positioning training system in coastal, but hot climate areas exacerbated the heat wave conditions even further with the monitored effects to cascading to plant primary metabolism.

### Plant Primary Metabolism

Stem water potential (Ψ_*stem*_) was greatly affected by doubling the amount of water applied. Customarily, vineyard operators would apply 40–50% of estimated ET_*c*_ in the study area (Torres et al., 2020). Contrary to our expectations, 80%ET_*c*_ did not incur higher stomatal conductance, transpiration or carbon assimilation for the instances measured. The relatively frequent (once a week) replacement of ET_*c*_ fractions could have allowed maintaining stomatal conductance, and ultimately yields, while water stored in the soil profile and stem water potential decreases through the season. However, it should not be discarded that in this study, more frequent gas exchange measurements, at a different time of the day and further from the irrigation day may have resulted in differences in stomatal conductance according to the amounts of water applied. Shade nets reduced the solar radiation by 40% that reached the sides of the first 0.25 m of the canopy (composed of shoots 1.4 m or longer) and this reduced carbon assimilation in those shaded leaves, but not the rest. The lower carbon assimilation measured in Shaded treatments resulted in lower pruning mass (Greer and Weedon, 2013). Plant organ mass (in our case dormant shoot mass) is an important indicator of photomorphogenesis. Its cell division and expansion may be altered by sun exposure through photomorphogenic effects at low rates (Robson et al., 2015). In addition, shade nets led to slightly better water status under 40% ET_*c*_, which could be indicative of a slightly lower water consumption by plants with nets covering the leaves around the fruit-zone. In studies where shading nets were placed above the canopy, soluble solids may be reduced through lower carbon assimilation rates when shriveling berries were not accounted (Greer and Weedon, 2013).

The application of 80% ET_*c*_ of water slightly increased berry and cluster mass. In fact, increases in berry size by both shade nets double irrigation were coupled to a reduction in TSS. Decreases in berry size and/or increases in berry TSS were observed in response to solar exposure by fruit-zone leaf removal (Poni et al., 2009). Solar overexposure may exacerbated transpiration loss in the fruit-zone, which has showed to affect cell expansion during leaf growth (Devi et al., 2015). However, in the last weeks of fruit development, higher dehydration/cell death may better explain the lower berry weight of Exposed–40%ET_*c*_ (Bonada et al., 2013; Caravia et al., 2016). Changes in berry size are often one of the first events in a concomitant effect, in which berry soluble solids are concentrated, enhancing other processes intertwined to hexose signaling, transport and metabolism (Dai et al., 2014; Rienth et al., 2016). The Exposed–40%ET_*c*_ berries, had higher TSS (+ 2°Brix) than the other treatments. However, berry acidity was mildly impacted and no significant effects were evident at harvest. Having similar titratable acidity values in berries exposed to higher temperatures was a result that was somewhat unexpected as warmer berry temperatures are associated with a higher respiration rate of malic acid (Sweetman et al., 2014). Contrarily to experiments in which ambient temperature was manipulated (e.g., open top chambers) that have a very homogeneous effect on fruit metabolism, overexposure is an extreme—although greatly patchy—factor depending on the position of each cluster and how these are covered by leaves and the time of the day. Therefore, the spatiotemporal variation in cluster temperature differences among treatments can be anything from similar (e.g., at night or clusters occluded by leaves) to + 12°C (i.e., at 1530 h in Southwest exposed clusters). This finding provided evidence that the greater berry temperature experienced by berries of Exposed vines—only experienced by some clusters on the Southwest aspect for a period of less than 4 h—has a milder impact on overall grape must pH and TA (Zarrouk et al., 2016) than persistent changes in ambient temperature reported elsewhere (Sweetman et al., 2014; Rienth et al., 2016; Torres et al., 2020).

### Plant Secondary Metabolites

Berry skin anthocyanins were initially greater in Exposed–40%ET_*c*_ 86 DAF but higher in the two shaded treatments (regardless of irrigation) during the last weeks prior to harvest. Whereas part of these results responded to a shift in berry development, mediated by the higher TSS of Exposed-40%ET_*c*_ grapevines, the lower concentration of anthocyanin of Exposed grapevines at harvest responded to degradation. This result is corroborated by Torres et al. (2020) where optimal solar radiation exposure for Cabernet Sauvignon grape berry is in fact, less than 20% of the global radiation reaching the cluster. Furthermore, the first visual symptoms of berry color blanching were observed on 17 August, not long after veraison and during the first two heat waves of the year (Figure 3). At 102 DAF, before the 4-day heat wave, anthocyanin concentration was at its maximum. However, when anthocyanins were measured 4 days after this heat wave, anthocyanin concentration decreased at variable rates in each, and all treatments (Table 2). Doubling the irrigation amount did not change anthocyanin degradation rates in any case, thus only the factor of Shading was presented. Studies dealing with the effects of light and temperature on anthocyanins have proposed reduced synthesis (Yamane et al., 2006), chemical (Mori et al., 2007) and biological degradation (Sarni-Manchado et al., 1997) of these. For instance, anthocyanin degradation rates were 20–23% in Shaded treatments, which was interpreted as an effect of ambient temperatures above 40°C on berries at a stage (102 DAF) that synthesis was most certainly stopped (Castellarin et al., 2007). Exposed grapes also experienced extreme berry temperatures beyond 50°C for a few hours, and nearly doubled anthocyanin degradation rates (30–38%). Isothermal degradation kinetics studies with fruit extracts indicated half-life values of few days at 50°C (Cemeroğlu et al., 1994), which were enough to corroborate to a great extent the anthocyanin degradation rates in the present study.

Malvidin was the most abundant anthocyanin in all samples and its proportion increased in detriment of the other 4 anthocyanins as ripening progressed. Changes in anthocyanin profile have been reported before and attributed to differential synthesis concomitant to changes in expression of flavonoid hydroxylases (Castellarin et al., 2006; Martínez-Lüscher et al., 2014). A differential degradation of each of the anthocyanins could also explain this result based on the different antioxidant capacity of each compound (Arroyo-Currás et al., 2016; Csepregi and Hideg, 2018). However, the increase in the proportion of malvidin increased at a constant pace, regardless of net synthesis or degradation (i.e., before or after 102 DAF) and (Torres et al., 2020) reported similar findings in Cabernet Sauvignon to further corroborate these results.

Inhibition of flavonol synthesis is not as sensitive to temperature as anthocyanin synthesis (Mori et al., 2005). In contrast, flavonol synthesis is mainly regulated by the exposure to UV-B radiation (Martínez-Lüscher et al., 2014). Flavonols accumulate in the outer layers of plant tissues screening a great part of solar UV radiation. The involvement of flavonols in the signaling and alleviation of oxidative stress was suggested (Agati et al., 2013; Watkins et al., 2017). However, our results do not provide strong evidence that flavonols may help to cope with high temperatures witnessed during heat waves. Therefore, when grape berries under field conditions are exposed to solar radiation with a subsequent increase in fruit temperature, there was a net synthesis flavonols up to mid/high levels of exposure (∼48% canopy porosity). However, their concentration then rapidly decrease as berries become overexposed with the heat waves (Torres et al., 2020). Our results presented herein corroborated this finding, where grapes from Exposed and Shaded plants reached similar concentrations of flavonols at harvest through two different levels of exposure. The behavior of berries were statistically separated as such in the Exposed thorough higher rates of biosynthesis; and then degradation, and in the Shaded, through lower rates of biosynthesis. Furthermore, Exposed berries only displayed a 17–27% decrease in flavonols. The berries in the Shaded treatments did not experience a decrease in flavonols during the heat wave, providing evidence that flavonols have a slightly higher stability than anthocyanins under high temperature. This differential net synthesis/degradation left a footprint in flavonol profile, increasing the proportion of kaempferol and quercetin, in detriment of myricetin and isorhamnetin as reported previously (Pastore et al., 2017; Martínez-Lüscher et al., 2019a; Torres et al., 2020).

### Heat Wave and Exposure Damage

In addition to the changes in chemical composition in the remaining fruit, a substantial part of the berries were completely mummified (at least 10%), and therefore, not sampled. During commercial harvest either through hand picking or with mechanical picking with on-board sorting, clusters and berries may be sorted and the grower may discard up to a 25% of the yield of this trial to optimize quality as desiccated berries may influence wine characteristics (Bonada et al., 2013). At the tissue level, the process of cell death has been characterized under elevated temperature and irrigation deficits (Bonada et al., 2013). It seems plausible that in our work, tissue temperatures of at least 50°C (berry temperature measurements while air temperature was 43.4°C were up to 54°C; data not shown) were enough to induce cell death, either progressively (Supplementary Figures S2E,F), or suddenly (Supplementary Figure S2G). However, to the best of our knowledge the estimation of certain temperature or irradiation thresholds for berry sunburn remain unexplored. In addition, cultural and environmental factors, featuring row orientation, trellis and training system, irrigation, and pre-exposure, may affect the incidence of sunburn for a given air temperature and irradiation (Webb et al., 2010; Zarrouk et al., 2016; Torres et al., 2020). Irrigating prior to an extreme heat event is meant by vineyard operators to increase leaf transpiration, leading to a cooling off effect at the fruit zone similar to overhead irrigation with sprinklers or misting system (Kliewer and Schultz, 1973). In our results, we had slightly higher berry temperatures in Exposed–40%ET_*c*_ than in Exposed–80%ET_*c*_ during the warmest part of the day on 114 DAF, but this was not associated to a reduced incidence of sunburn, less anthocyanin degradation or plant fitness. As both irrigation amounts tested in this study had similarly high transpiration rates, it still remains to be tested whether a under low transpiration rates, misting could have ameliorated berry temperatures. Before these results may be extrapolated, it must be noted that vertically shoot positioned trellis systems, compared to sprawling training systems, have few leaf layers around the fruit zone, and thus, increases in transpiration may not influence berry temperature as much. Therefore, the potential benefit of irrigation prior to extreme heat events should not be completely discarded.

## Conclusion

After a great number of studies—mostly performed in cooler climates—reporting the beneficial effects of solar radiation and water deficits for fruit ripening and especially flavonoids biosynthesis, new research focus is needed about the detrimental effects of the extreme climatic events such as drought and heat waves. Partial shading can have a positive role in the retention of grape berry skin flavonoids in a scenario of high temperatures and reduced cloud-cover. Conversely, solar exposure did not have a great effect on must acidity as controlled increases in air temperature reported elsewhere. Direct exposure to solar radiation was a necessary cooperator together with extreme heat events to produce fruit damage as practically all the damage recorded in this study was found in Southwest aspects of canopies without shade nets. Doubling irrigation amount had some mild effects lowering berry temperature and avoiding berry dehydration during the last part of ripening. Although doubling the amount of irrigation used may not be justified by this little gain, some additional irrigation prior to the heat events may be of practical use in rain-fed vineyards or with minimal irrigation in a case such as this. This study provided evidence of the necessity of field studies performed during extreme weather events, such as heat waves, to help complement the information gained under constant elevated temperature for the adaptation of cropping systems to climate change.

## Data Availability Statement

The raw data supporting the conclusions of this article will be made available by the authors, without undue reservation.

## Author Contributions

SK acquired the funding. CC collected the field data. JM-L and LB oversaw the field and lab data collection. JM-L wrote the first version of the manuscript. All authors contributed to the article and approved the submitted version.

## Conflict of Interest

The authors declare that the research was conducted in the absence of any commercial or financial relationships that could be construed as a potential conflict of interest.

## Supplementary Material

Supplementary Figure 1 Installation of Black-40 shade net 1 m (w) × 6 m (long) along the fruit zone of the experimental plot **(A)**. Installed Black-40 shade net on the Northeast side of the fruit zone of the experimental along the experimental vines **(B)** In-row spacing: 2.0 m, row spacing 2.4 m.

Supplementary Figure 2 Appearance of Exposed treatment clusters on the Southwest aspect of canopy **(A,B)**. Clusters on the Southwest aspect of the canopy of Shaded plants with the nets on **(C)** and same clusters after removing the shade nets **(D)**. Initial damage on clusters few weeks after veraison **(E)** and progression of the damage through harvest **(F)**. Sudden berry abortion observed on during the heat wave (104 DAF; 1 September) **(G)**.
