# Supplementary figures and images for: Mitigating Heat Wave and Exposure Damage to “Cabernet Sauvignon” Wine Grape With Partial Shading Under Two Irrigation Amounts
Source: Front Plant Sci. 2020 Nov 10;11:579192. doi: 10.3389/fpls.2020.579192 (PMC7683524; doi:10.3389/fpls.2020.579192)

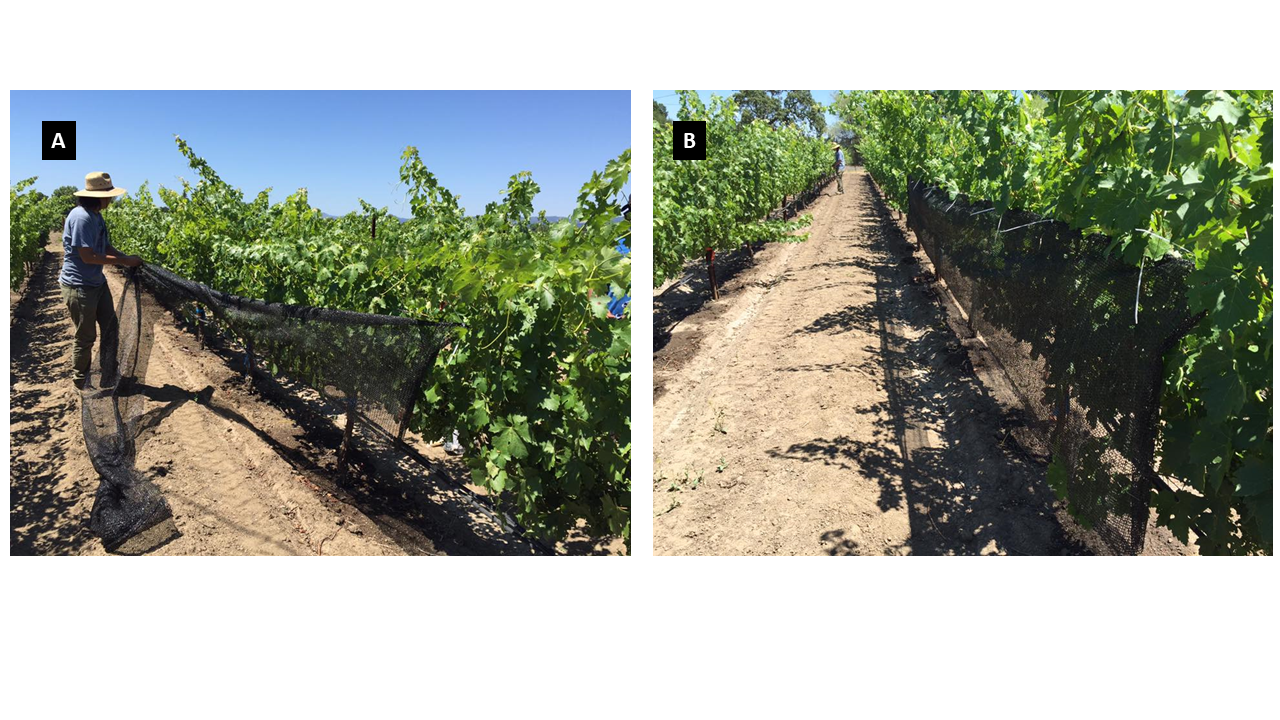

Supplement: Supplementary Figure 1 — Installation of Black-40 shade net 1 m (w) × 6 m (long) along the fruit zone of the experimental plot (A). Installed Black-40 shade net on the Northeast side of the fruit zone of the experimental along the experimental vines (B) In-row spacing: 2.0 m, row spacing 2.4 m. [file Image_1.TIF]

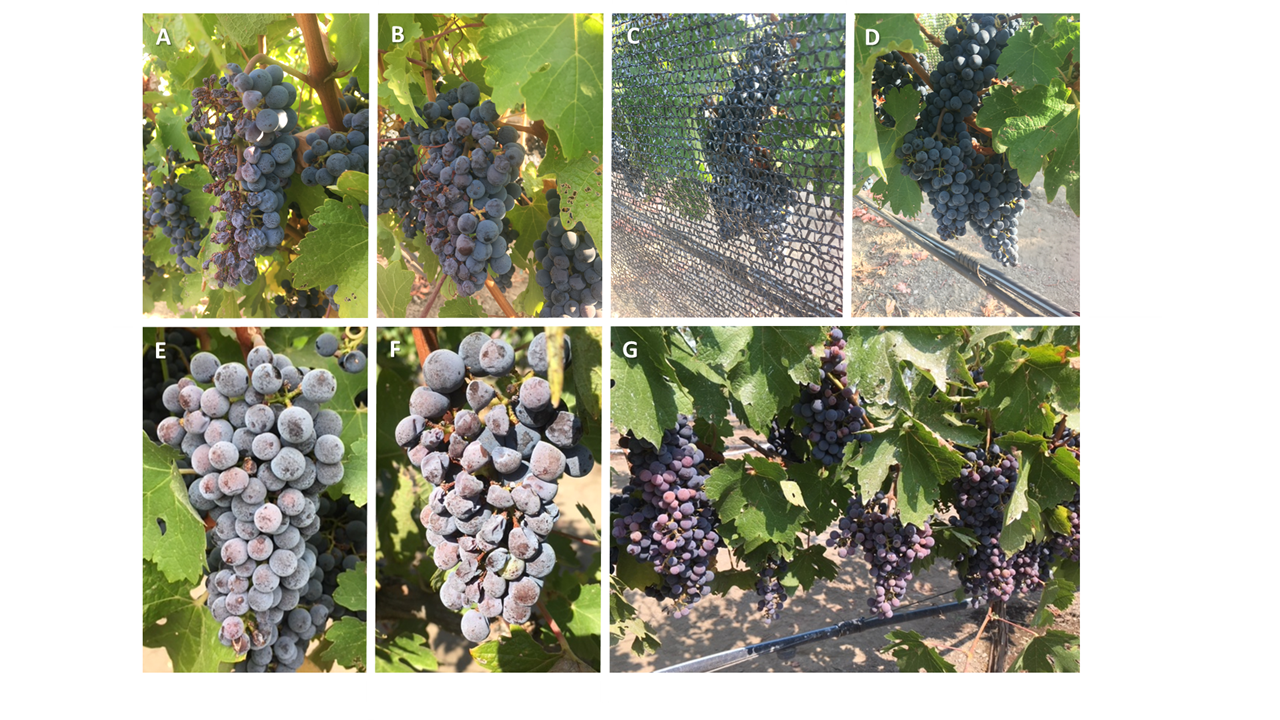

Supplement: Supplementary Figure 2 — Appearance of Exposed treatment clusters on the Southwest aspect of canopy (A,B). Clusters on the Southwest aspect of the canopy of Shaded plants with the nets on (C) and same clusters after removing the shade nets (D). Initial damage on clusters few weeks after veraison (E) and progression of the damage through harvest (F). Sudden berry abortion observed on during the heat wave (104 DAF; 1 September) (G). [file Image_2.TIF]
